# Commentary: *Drosophila* GATA Factor Serpent Establishes Phagocytic Ability of Embryonic Macrophages

**DOI:** 10.3389/fimmu.2018.01582

**Published:** 2018-07-06

**Authors:** Susanna Valanne, Laura Vesala, Mika Rämet

**Affiliations:** ^1^Laboratory of Experimental Immunology, Faculty of Medicine and Life Sciences, BioMediTech Institute, University of Tampere, Tampere, Finland; ^2^PEDEGO Research Unit, Medical Research Center Oulu, University of Oulu, Oulu, Finland; ^3^Department of Children and Adolescents, Oulu University Hospital, Oulu, Finland

**Keywords:** phagocytosis, *Drosophila melanogaster*, apoptosis, innate immune response, plasmatocyte

Phagocytosis of particles by cells is an ancient, evolutionarily highly conserved process. It is essential for normal development, tissue homeostasis, and immunity in a wide range of organisms from flies to man. In *Drosophila melanogaster*, plasmatocytes, the most abundant blood cell type, participate both in the elimination of apoptotic corpses during development (1, 2) and in the receptor-mediated phagocytosis of microbes, an indispensable process for immunity (3–5).

The ability of phagocytic cells to recognize and internalize particles is based on the expression of various phagocytic receptors. In *D. melanogaster*, the key receptors for recognition of both apoptotic corpses (Six-Microns-Under, Draper, Croquemort) and microbes (Eater, Scavenger receptor-CI, Nimrod) are rather well described (3, 6–11).

In the recent issue of Frontiers in Immunology, Shlyakhover and his co-workers (12) elegantly describe the central role of transcriptional regulator Serpent for the phagocytic ability of embryonic macrophages in *D. melanogaster*. Serpent is a GATA factor, which is shown by the authors to be both required and sufficient for the expression of phagocytic receptors needed for engulfment of apoptotic corpses in the embryonic macrophages. In the Serpent mutant embryos, phagocytosis of apoptotic corpses was severely impaired, and this was associated with a loss of *Six-Microns-Under, Draper*, and *Croquemort* expression. Furthermore, expression of any of these receptors partially rescued the phagocytosis deficiency in the Serpent mutants.

Thus, this recent study beautifully demonstrates the central role of Serpent as the master regulator of phagocytosis of apoptotic cells by controlling the expression of receptors required for recognition of these particles. However, it has been a long-standing observation that *serpent* expression—together with components of the transcriptional coactivator Mediator complex Med12–Med13—is also required for phagocytosis of microbes in macrophage-like, *D. melanogaster* embryo-derived S2 cells (13, 14) (Figure 1). Serpent was identified in the very first high-throughput RNA interference (RNAi) screen as a regulator of bacterial cell surface binding and phagocytosis (13). RNAi targeting *serpent* reduced phagocytosis of heat-killed, FITC-labeled *Escherichia coli* (phagocytic index 21 ± 11 of controls) and *Staphylococcus aureus* (phagocytic index 20 ± 6 of controls) (3). Therefore, Serpent appears to control the expression of cell surface proteins responsible for microbial binding and uptake in addition to receptors required for the recognition of apoptotic corpses (Figure 1). In fact, by performing a transcriptional analysis of S2 cells in which *serpent* was knocked down by RNAi (3, 14), 46 genes were identified with signal sequence and/or transmembrane domain whose expression was downregulated by more than twofold after *serpent* RNAi (3). These included known phagocytic receptors for microbes (*Eater* and *Scavenger receptor-CI*) indicating that Serpent is a master regulator of also microbial pattern recognition in phagocytosis. Of note, our analysis of Serpent-dependently expressed genes revealed also *Six-Microns-Under* (also called *Nimrod C4* and *CG16876*) as one of the genes expressed in Serpent-dependent manner.

**Figure 1:**
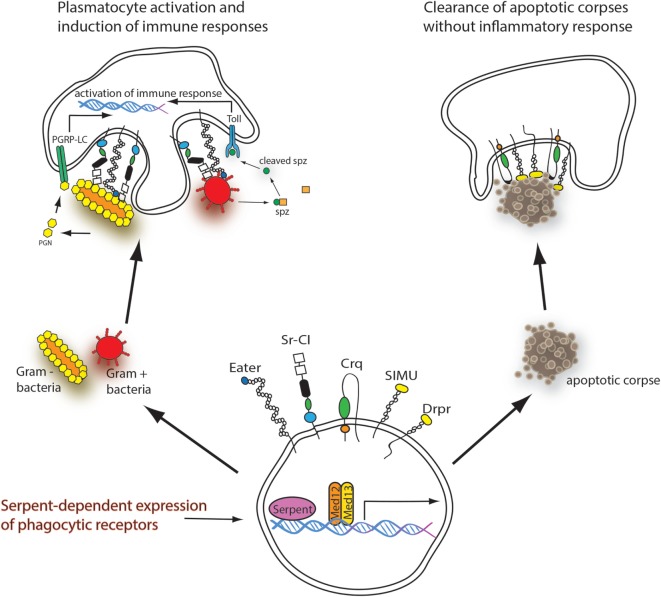
Serpent-dependent expression of phagocytic receptors. The naïve hemocyte expresses both apoptotic and immune-related phagocytosis receptors in a Serpent and Med12/Med13-dependent manner. Upon recognition of “eat me” signals produced by apoptotic corpses, the receptors Croquemort (Crq), Draper (Drpr), and Six-Microns-Under (SIMU) bind to the apoptotic corpse and initiate its phagocytosis and degradation without inflammatory response (right). When the hemocyte meets bacteria, the phagocytic receptors Eater and scavenger receptor CI (Sr-CI) bind to the bacteria initiating phagocytosis. Bacteria also induce systemic and cellular immune responses *via* the Imd and the toll pathways (left).

Altogether, these findings elaborate the importance of GATA factor Serpent in transcriptional control of the overall phagocytic competence of macrophage-like cells in *D. melanogaster*. As the same transcription factor controls the expression of genes necessary for clearance of particles without inflammatory response as well as immune response associated receptors, it seems that in *Drosophila*, professional phagocytic cells possess capability for both anti- and pro-inflammatory responses depending on the cargo they recognize. Upon immune activation, *Drosophila* hemocytes produce antimicrobial peptides (15) and change their morphology (16, 17). This resembles polarization of mammalian macrophages toward a “pro-inflammatory” M1 phenotype by exposure to lipopolysaccharide together with TH1 cytokine IFN-γ. As a consequence, M1 polarized macrophage is an effector cell in TH1 cellular immune responses, whereas the alternatively activated M2 macrophage has immunosuppressive properties, for example, in wound healing and tissue repair. In contrast to mammals, *Drosophila* lacks interferon-γ and adaptive immunity, so there must be alternative means to direct plasmatocyte polarization. With numerous advantageous genetic tools together with efforts to define hemocyte lineages using *in vivo* hemocyte reporters (16), *D. melanogaster* will continue to be an exciting model to study factors affecting differentiation of hemocyte lineages and mechanisms controlling phagocytic competence and activation of plasmatocytes.

## Author Contributions

All authors wrote and approved the final version of the manuscript.

## Conflict of Interest Statement

The authors declare that the research was conducted in the absence of any commercial or financial relationships that could be construed as a potential conflict of interest.
